# Supplementary material for: Unveiling the structural spectrum of SARS-CoV-2 fusion by in situ cryo-ET
Source: Nat Commun. 2025 Jun 3;16:5150. doi: 10.1038/s41467-025-60406-z (PMC12134289; doi:10.1038/s41467-025-60406-z)
Supplement: Supplementary file 1 — Supplementary information [file 41467_2025_60406_MOESM1_ESM.pdf]

SUPPLEMENTAL DATA

Supplementary Table 1. Cryo-ET data acquisition statistics

Sample	Virion only	Virion + VLP_L	Virion+ VLP_H	Virion+ Trypsin	Virion + VLP_L + Trypsin	Virion + VLP_H + Trypsin	Virion + VLP_L + Trypsin
**Microscope**	Titan Krios	Titan Krios	Titan Krios	Titan Krios	Titan Krios	Titan Krios	Titan Krios
**Voltage (keV)**	300						
**Energy-filter (eV)**	20	20	20	20	20	20	20
**Detector**	TFS Selectris X and Falcon 4i	Gatan Quantum K3 Direct Electron Detector	Gatan Quantum K3 Direct Electron Detector	TFS Selectris X and Falcon 4i	Gatan Quantum K3 Direct Electron Detector	Gatan Quantum K3 Direct Electron Detector	TFS Selectris X and Falcon 4i
**Pixel size (A)**	1.501	1.34	1.34	1.501	1.34	1.34	1.501
**Defocus range (μm)**	-2.5 to -3.5	-2.5 to -3.5	-2.5 to -3.5	-2.5 to -3.5	-2.5 to -3.5	-2.5 to -3.5	-2.5 to -3.5
**Defocus increment (μm)**	0.3	0.3	0.3	0.3	0.3	0.3	0.3
**Acquisition scheme**	-60°/60°, 3° dose symmetric	-60°/60°, 3° dose symmetric	-60°/60°, 3° Dose symmetric	-60°/60°, 3° Dose symmetric	-60°/60°, 3° dose symmetric	-60°/60°, 3° dose symmetric	-60°/60°, 3° dose symmetric
**Total Dose (electrons/A2)**	123	123	123	123	123	123	123
**Frame number**	10	10	10	10	10	10	10
**Tomogram number**	8	51	56	21	50	50	70

Supplementary Table 2. Cryo-ET data acquisition and subtomogram averaging statistics for WS6.

Sample	Virion + WS6	Virion + WS6 + Trypsin	Virion + WS6 + VLP_L	Virion + WS6 + VLP_L + Trypsin
**Data acquisition**				
Microscope	Titan Krios	Titan Krios	Titan Krios	Titan Krios
Voltage (keV)	300			
Energy-filter	Yes	Yes	Yes	Yes
Slit width (eV)	20	20	20	20
Detector	TFS Selectris X and Falcon 4i	Gatan Quantum K3 Direct Electron Detector	TFS Selectris X and Falcon 4i	TFS Selectris X and Falcon 4i
Pixel size (A)	1.501	1.34	1.501	1.501
Defocus range (µm)	-2.5 to -3.5	-2.5 to -3.5	-2.5 to -3.5	-2.5 to -3.5
Defocus increment (µm)	0.3	0.3	0.3	0.3
Acquisition scheme	-60°/60°, 3° dose symmetric	-60°/60°, 3° dose symmetric	-60°/60°, 3° dose symmetric	-60°/60°, 3° dose symmetric
Total Dose (electrons/A2)	123	123	123	123
Frame number	10	10	10	10
Tomogram number	36	19	30	36
**Image processing**				
	With WS6		Without WS6	
No. of subtomograms	1,398		963	
Resolution at 0.143 FSC (Å)	9.3		8.3	
b-factor applied	-50		-50	
Symmetry applied	C3		C3	
Data deposition	EMD-51333		EMD-51334	

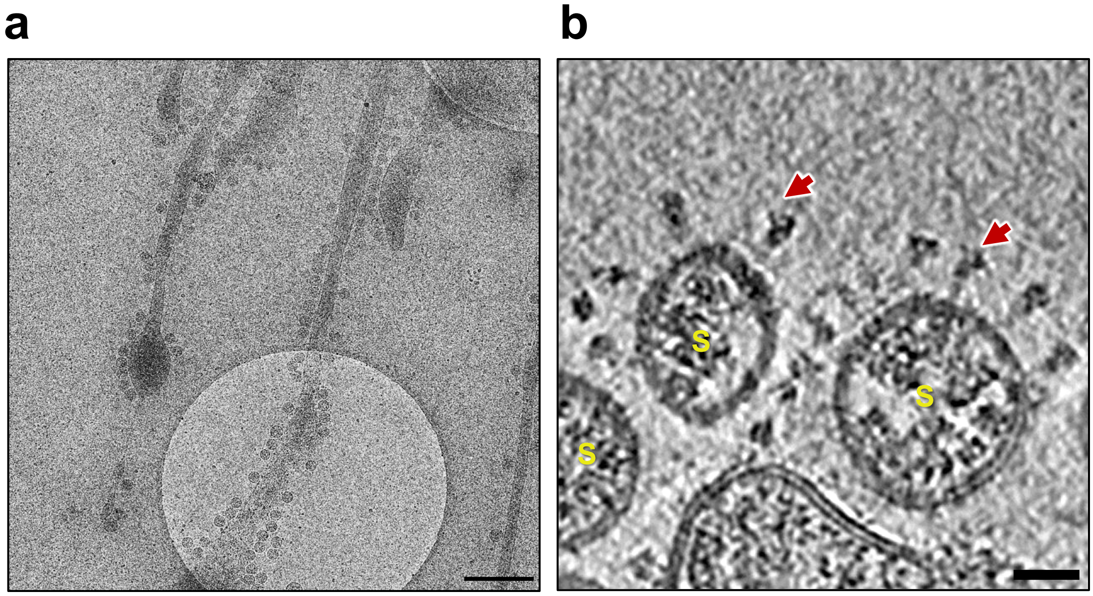

Supplementary Figure 1: Characterization of egressed SARS-CoV-2 virions from Vero cells. (a) Micrographs of egressed SARS-CoV-2 virions on the EM grid, in the context of infected Vero cells. (**b**) A tomographic slice displaying an enlarged view of the egressed virions. Red arrows indicate prefusion spikes. SARS-CoV-2 virions are labelled “S”. Scale bars: 500 nm (a) and 20 nm (b).

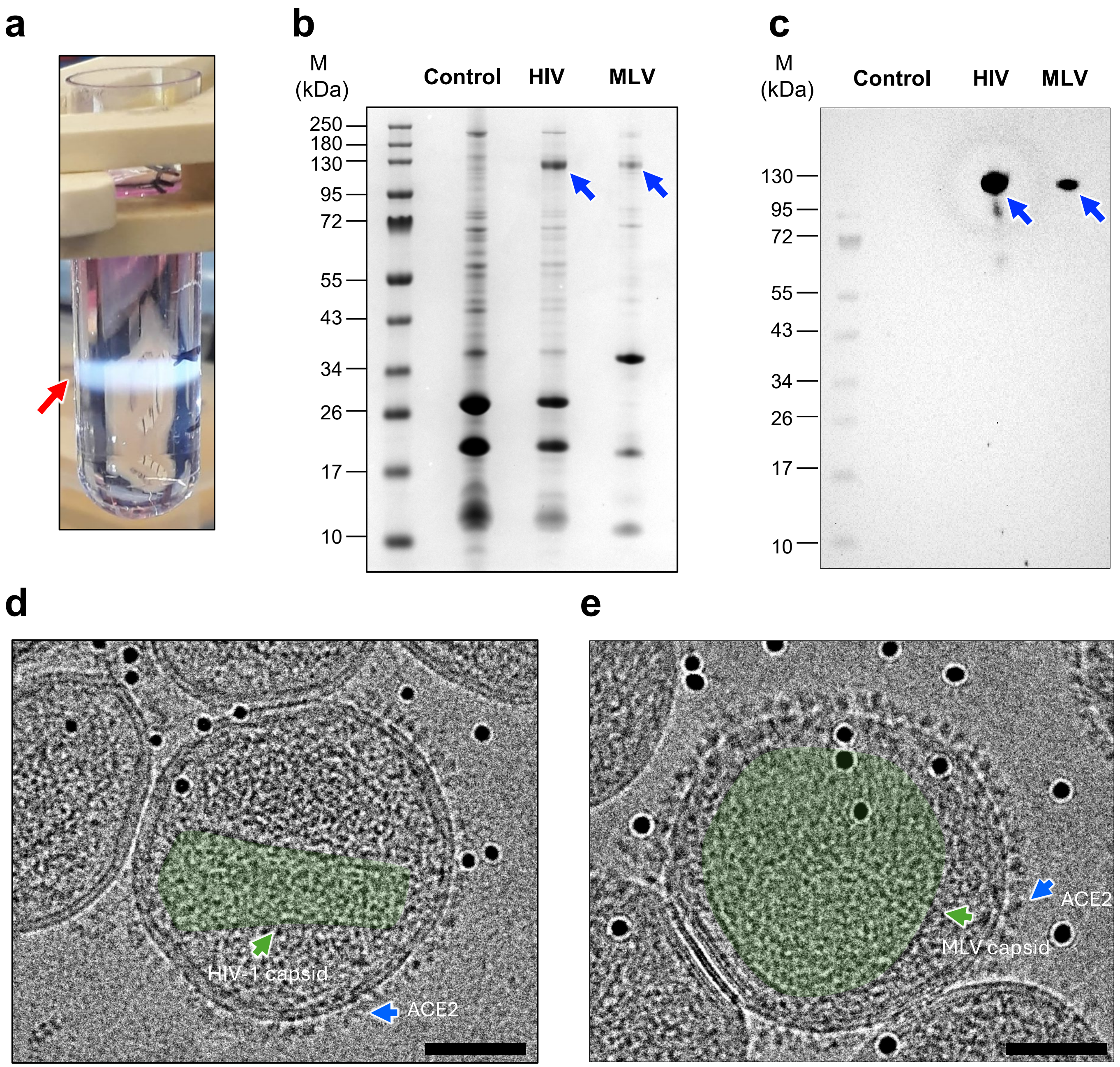

Supplementary Figure 2: ACE2 expressing HIV-1 VLP Production, Purification, and Validation. (a) OptiPrep™ density gradient after ultracentrifugation, showing the band corresponding to ACE2/HIV-1 VLPs (red arrow). (**b**) SDS-PAGE gel analysis of VLPs: control HIV-1 VLPs (without ACE2), ACE2/HIV-1 VLPs, and ACE2/MLV VLPs. (**c**) Western blot analysis of VLPs using monoclonal anti-ACE2 antibody (ProteinTech 21115-1-P; diluted 1:5,000), confirming the presence of ACE2 in HIV-1 and MLV VLPs. ACE2 bands are indicated by blue arrows. (**d-e**) Transmission electron micrographs of ACE2/HIV-1 VLPs **(d)** and ACE2/MLV VLPs **(e)**. HIV-1 and MLV capsids are indicated by green arrows, and ACE2 dimers are highlighted by blue arrows. Scale bars: 50 nm.

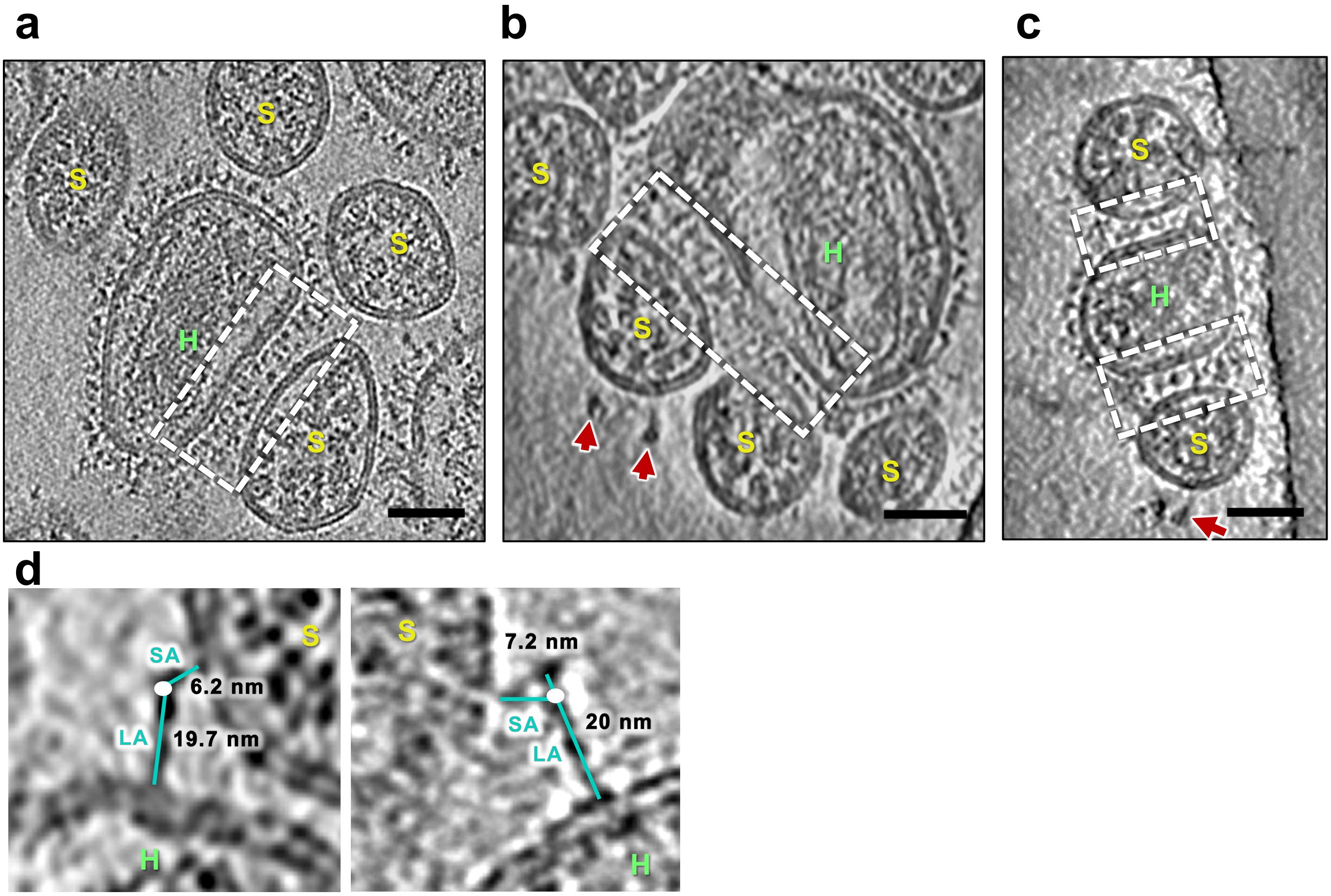

Supplementary Figure 3: Interaction of SARS-CoV-2 virions with $ACE2_{VLP}$s. (a-c) Examples of cluster binding mode at membrane-membrane interfaces between SARS-CoV-2 virions and $ACE2_{VLP}$s (dashed white boxes). Red arrows indicate prefusion spikes. SARS-CoV-2 virions are labelled "S" and $ACE2_{VLP}$s are labelled "H." Scale bars: 50 nm. (**d**) Measurements of the shorter arm (SA) and longer arm (LA) of the partial backfolding intermediate densities.

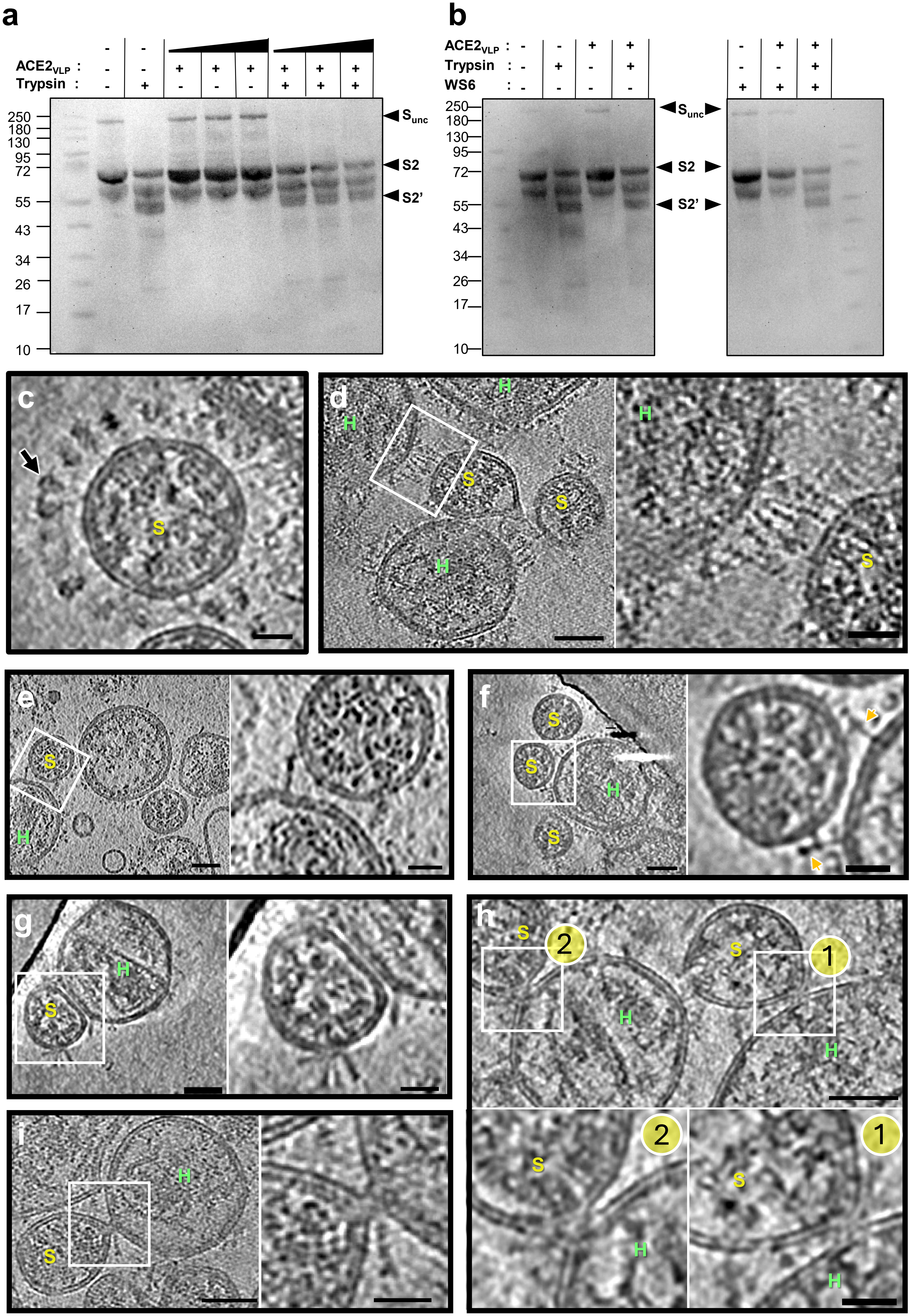
a
ACE2VLP :
Trypsin :
250
180
130
95
72
55
43
34
26
17
10
Sunc
S2
S2'
b
ACE2VLP :
Trypsin :
WS6 :
c
d
e
f
g
h
i
S
H
1
2

Supplementary Figure 4: Cleavage of S2’ by trypsin. (**a**) Western blot analysis of SARS-CoV-2 S2’ cleavage by trypsin in the presence and absence of ACE2 VLPs, using a monoclonal anti-S2 antibody (Thermo Fisher Scientific MA5-35946). (**b**) Western blot analysis of S2’ cleavage by trypsin in the presence and absence of the WS6 antibody, using a monoclonal anti-S2 antibody (Thermo Fisher Scientific MA5-42384). In (**a**), different concentrations of ACE2 VLPs were used: 0.025 mg/ml, 0.1 mg/ml, and 0.4 mg/ml (from left to right). The uncleaved spike protein (S-unc), S2, S2’ cleavage products. Western blot experiments in (a) and (b) were independently repeated three times with similar results. **(c)** A tomographic slice of a trypsin-treated virions, showing prefusion spikes (red arrow). **(d-i)** Tomographic slices (left) and enlarged views of the boxed areas (right) showing different fusion intermediates: extended intermediate (**d**), partial backfolding intermediate (**e**), further partial backfolding intermediate (**f**), tightly opposing phase (**g**), dimpling phase (h-1), hemifusion (h-2) and initial pore (i). ACE2 VLPs are labeled "H," and SARS-CoV-2 virions are labeled "S." Scale bars: 50 nm (d–g, left) and 20 nm (d–g, right).

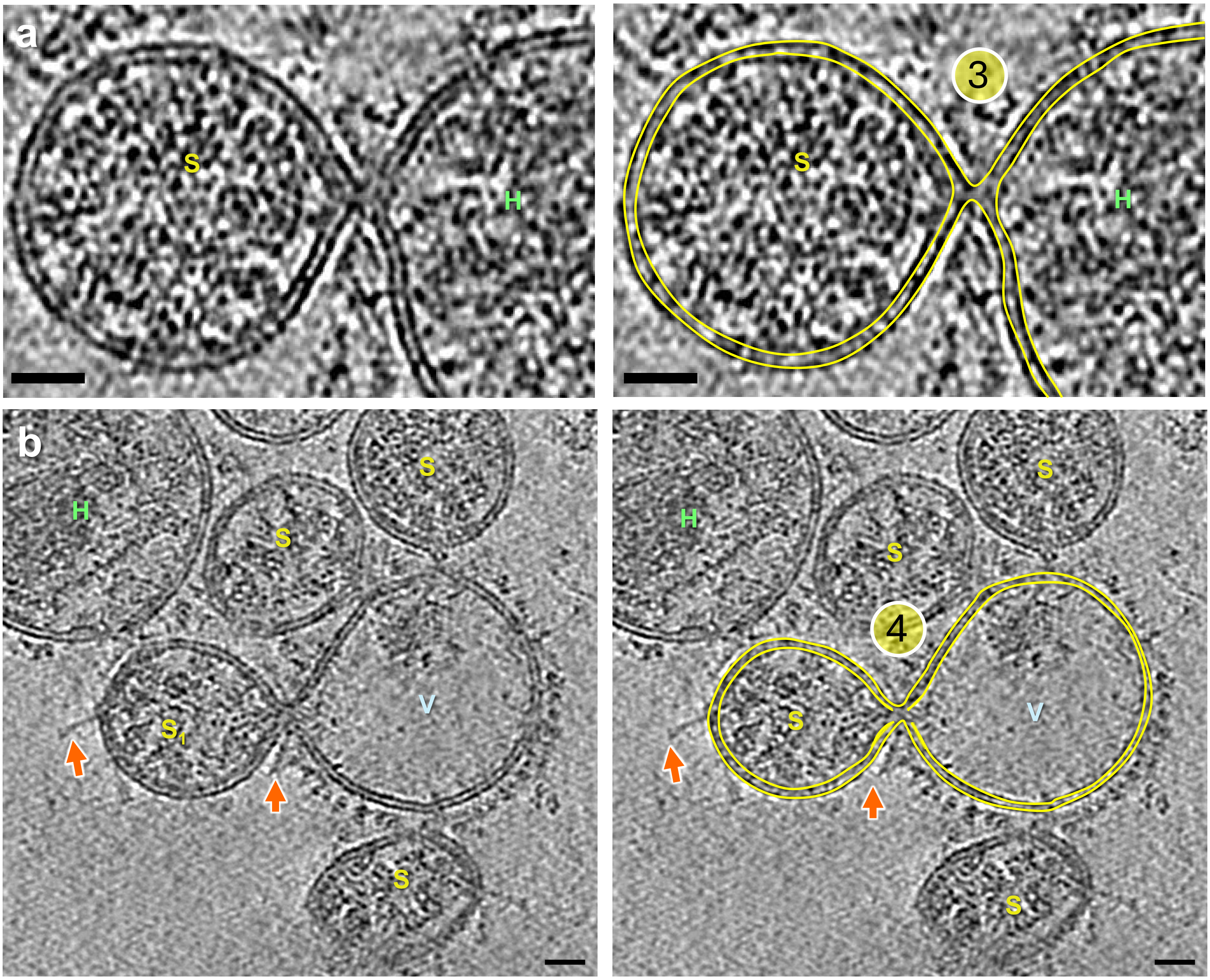

Supplementary Figure 5: Hemifusion and Initial fusion pore formation. **(a)** A hemifusion site (site 3) is shown in a raw tomographic slice (left), with the corresponding segmentation outlined. **(b)** A vesicle with ACE2 dimers on its surface is surrounded by multiple SARS-CoV-2 virions. The vesicle interacts with a virion (S_1) and forms an initial pore (site 4), as shown in a tomographic slice (left) and with membrane segmentation highlighting the leaflets of the bilayer (right, yellow outlines). SARS-CoV-2 virions are labelled “S,” the ACE2-expressing vesicle labelled “V,” and $ACE2_{VLP}$ is labelled “H.” The postfusion spikes are indicated with orange arrows. Scale bars: 20 nm.

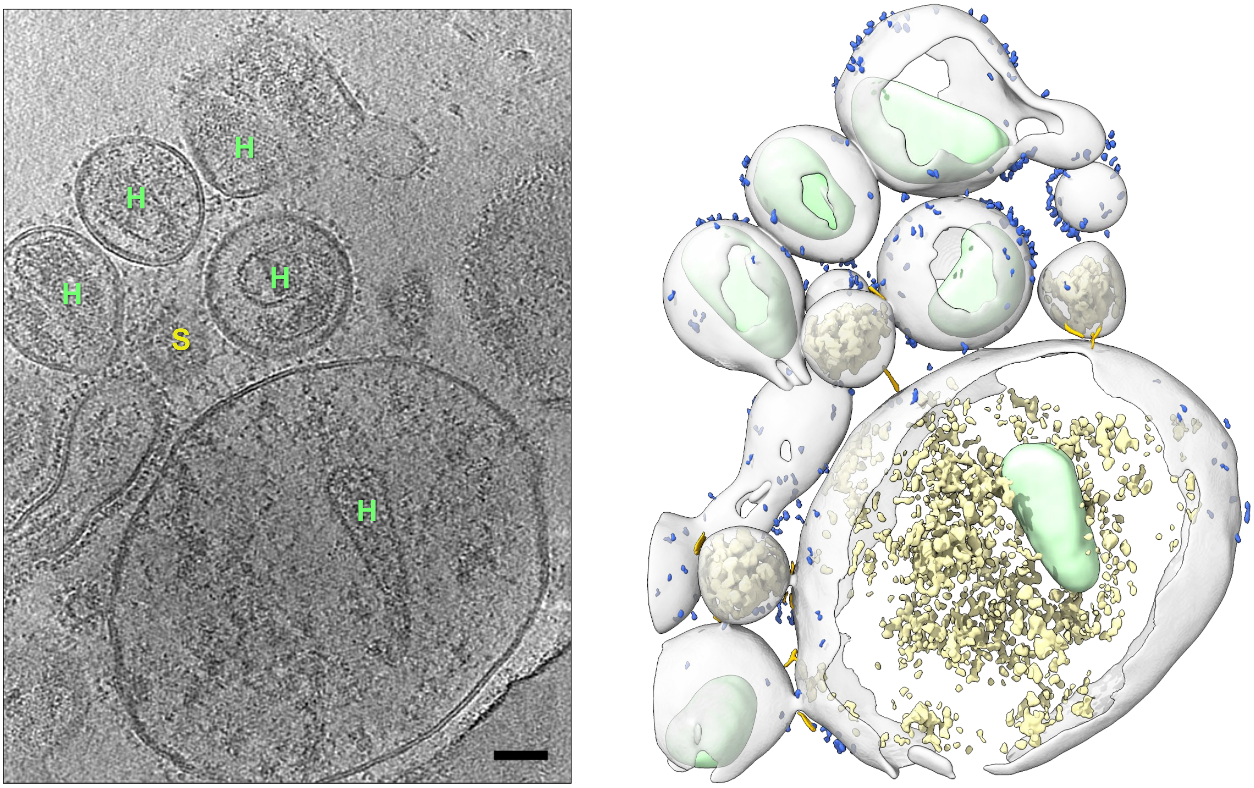

Supplementary Figure 6: Multiple rounds of fusion between SARS-CoV-2 virions with $ACE2_{VLP}$s in the presence of trypsin. Formation of a giant HIV-1 core-containing particle resulting from fusion with multiple SARS-CoV-2 virions. Displayed are a tomographic slice (left) and the corresponding segmented volume (right). In the tomographic slice, a SARS-CoV-2 virion is labelled "S" and $ACE2_{VLP}$s are labelled "H." In the segmented volume, spike intermediates are in gold, ACE2 dimer in blue, and HIV-1 capsid in light green. Scale bar: 50 nm.

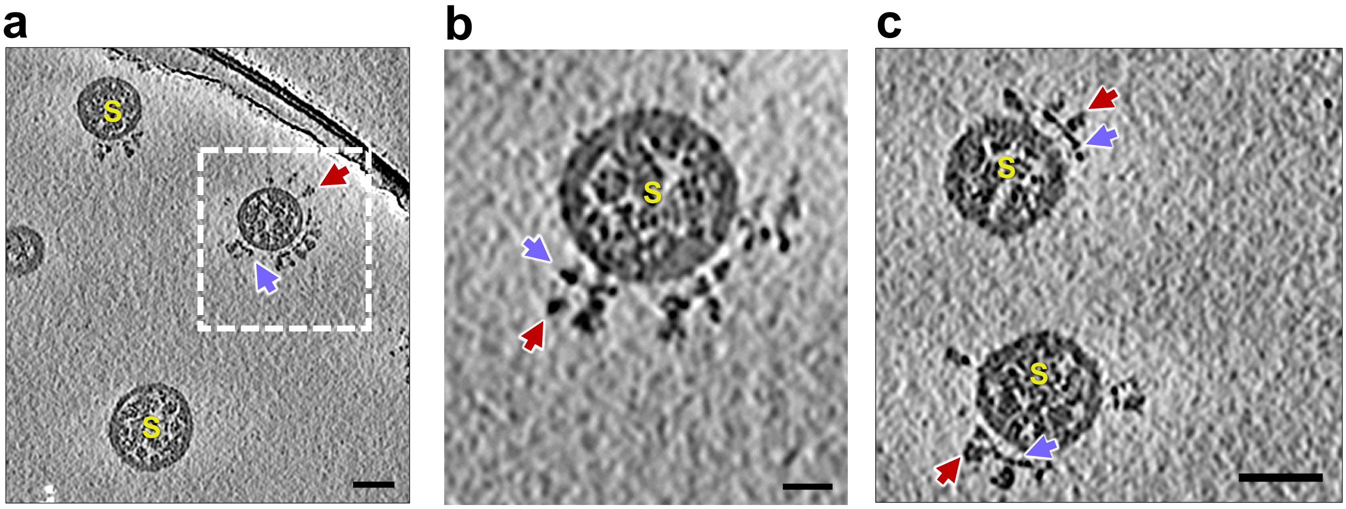

Supplementary Figure 7: Interaction between WS6 and SARS-CoV-2 spike. (a-c) Tomographic slices and enlarged view **(b)** of SARS-CoV-2 virions bound with WS6. Scale bars: 50 nm (a, c); 20 nm (b).

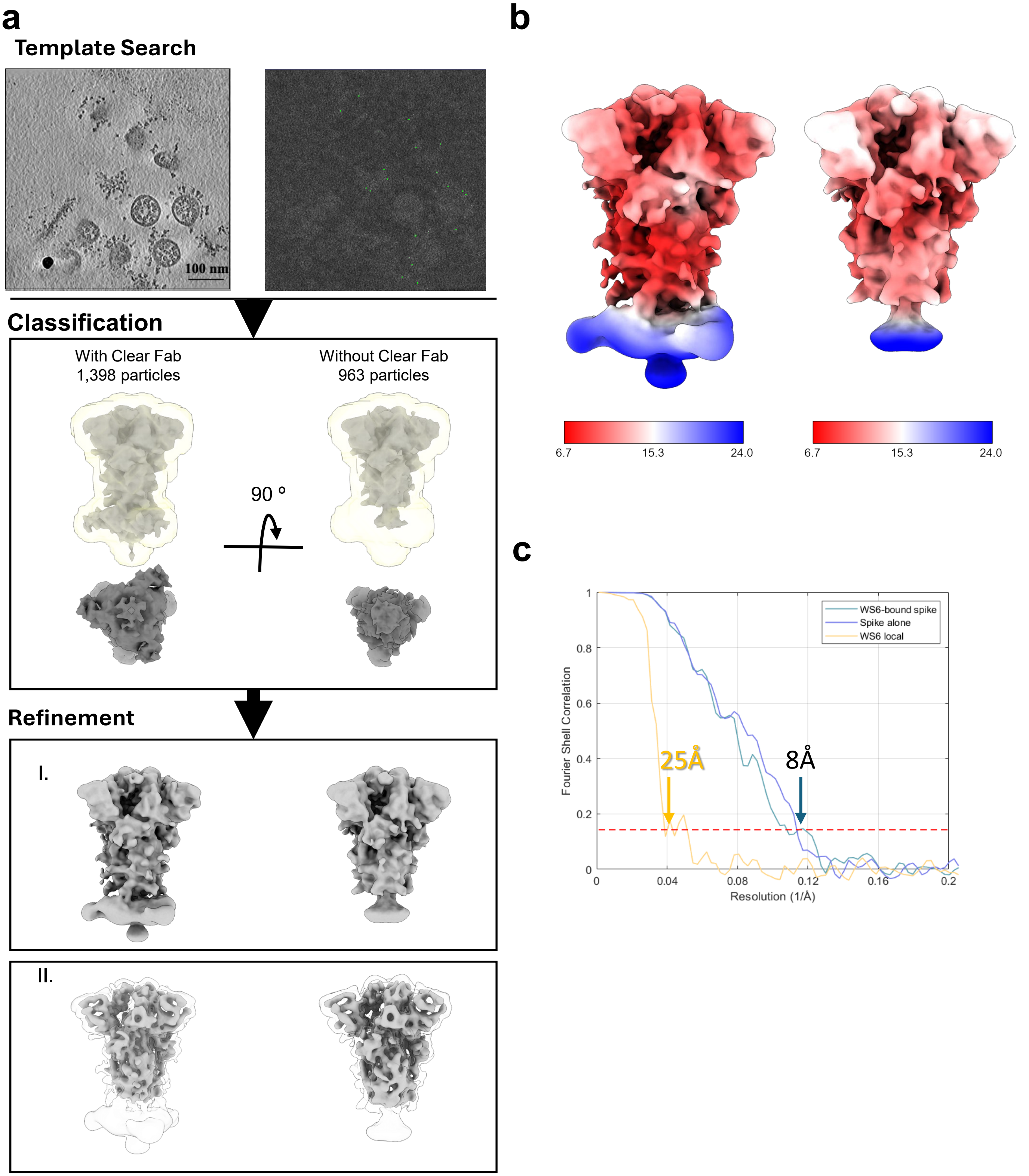

Supplementary Figure 8: Cryo-ET subtomogram averaging of S2 antibody WS6-bound native SARS-CoV-2 spikes on virions. (a) Data processing workflow, including template search, classification, and refinement (I. WS6-based threshold; II. Spike-based threshold map; Mask used for classification is shown in light yellow. **(b)** Cryo-ET subtomogram average maps, surface coloured by local resolution. (**c)** Fourier Shell Correlation (FSC) curves of subtomogram averages. The resolutions of spike alone, WS6-bound spike, WS6 local refinement are indicated at the 0.143 FSC cut-off.

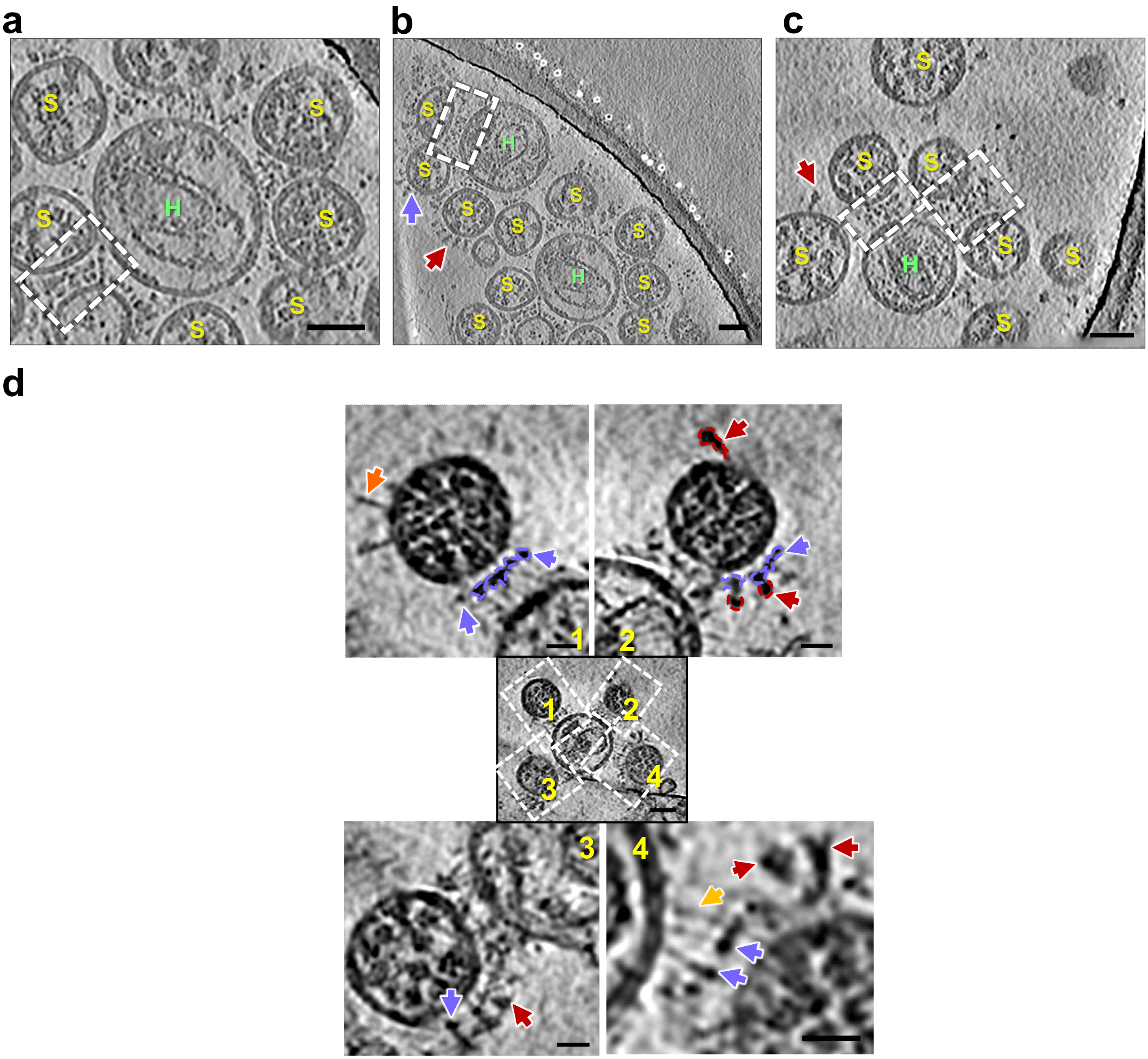

Supplementary Figure 9: Effect of S2 antibody WS6 on SARS-CoV-2 fusion. (a-c) Tomographic slices of SARS-CoV-2 virions bound with WS6, followed by incubation with $ACE2_{VLPs}$ and trypsin. Dashed boxes highlight membrane-membrane interfaces of SARS-CoV-2 virions and $ACE2_{VLPs.}$ Red arrows point to prefusion S spikes, and purple arrows highlight WS6. Scale bars, 50 nm in a-c. **(d)** Tomographic slices from Figure 4d display SARS-CoV-2 virions bound to the WS6 antibody, followed by incubation with $ACE2_{VLPs}$ and trypsin. Enlarged views, numbered **1-4**, highlight the densities corresponding to prefusion spikes (red arrows) and WS6 antibody (purple arrows). Extended fusion intermediates and postfusion spikes are indicated by gold and orange arrows, respectively. Scale bars represent 50 nm in the central image and 20 nm in the enlarged views surrounding it.
