# Supplementary material for: Unveiling the structural spectrum of SARS-CoV-2 fusion by in situ cryo-ET
Source: Nat Commun. 2025 Jun 3;16:5150. doi: 10.1038/s41467-025-60406-z (PMC12134289; doi:10.1038/s41467-025-60406-z)
Supplement: Supplementary file 2 — Description Of Additional Supplementary File [file 41467_2025_60406_MOESM2_ESM.pdf]

Description of Additional supplementary files

Supplementary Movies 1 and 2. Binding of multiple SARS-CoV-2 virions to one ACE2VLP.

Supplementary Movie 1 illustrates the interaction between three SARS-CoV-2 virions and a single ACE2VLP (corresponding to Figure 1b, c). The interface between the prefusion spikes of SARS-CoV-2 and ACE2 dimers predominantly exhibits a clustered binding pattern, with occasional single binding events observed Supplementary Movie 2 depicts the binding of five SARS-CoV-2 virions to a single ACE2VLP (Figure 1d). Scale bars: 50 nm. ACE2VLPs are labeled as "H" and SARS-CoV-2 virions as "S".

Supplementary Movie 3. Trypsin-induced tightly opposing phase (magenta arrow) and dimpling state (cyan arrow) of SARS-CoV-2 virions with ACE2VLP (Figure 3a).

In the segmented volumes, postfusion spikes are represented in orange, RNPs in yellow, ACE2 dimers in blue, and the HIV-1 capsid in light green

Supplementary Movie 4. Hemifusion (blue arrow) and initial fusion pore formation (brown arrow) (Figure 3b).

In the segmented volumes, postfusion spikes are shown in orange, RNPs in yellow, ACE2 dimers in blue, and the HIV-1 capsid in light green.

Supplementary Movie 5. Multiple fusion events between SARS-CoV-2 virions and a central ACE2VLP.

This movie (Figure 3c) demonstrates multiple fusion events involving four SARS-CoV-2 particles and a central ACE2VLP, providing visual evidence of the fusion process. Scale bar: 50 nm. ACE2VLPs are labeled as "H" and SARS-CoV-2 virions as "S".
