# Supplementary material for: Unveiling the structural spectrum of SARS-CoV-2 fusion by in situ cryo-ET
Source: Nat Commun. 2025 Jun 3;16:5150. doi: 10.1038/s41467-025-60406-z (PMC12134289; doi:10.1038/s41467-025-60406-z)
Supplement: Supplementary file 8 — Reporting summary [file 41467_2025_60406_MOESM8_ESM.pdf]

nature portfolio

Corresponding author(s): Peijun Zhang

Last updated by author(s): May 10, 2025

Reporting Summary

Nature Portfolio wishes to improve the reproducibility of the work that we publish. This form provides structure for consistency and transparency in reporting. For further information on Nature Portfolio policies, see our Editorial Policies and the Editorial Policy Checklist.

Statistics

For all statistical analyses, confirm that the following items are present in the figure legend, table legend, main text, or Methods section.

n/a	Confirmed	
☐	☒	The exact sample size (*n*) for each experimental group/condition, given as a discrete number and unit of measurement
☒	☐	A statement on whether measurements were taken from distinct samples or whether the same sample was measured repeatedly
☐	☒	The statistical test(s) used AND whether they are one- or two-sided<br>*Only common tests should be described solely by name; describe more complex techniques in the Methods section.*
☒	☐	A description of all covariates tested
☐	☒	A description of any assumptions or corrections, such as tests of normality and adjustment for multiple comparisons
☐	☒	A full description of the statistical parameters including central tendency (e.g. means) or other basic estimates (e.g. regression coefficient) AND variation (e.g. standard deviation) or associated estimates of uncertainty (e.g. confidence intervals)
☐	☒	For null hypothesis testing, the test statistic (e.g. F, t, r) with confidence intervals, effect sizes, degrees of freedom and P value noted<br>*Give P values as exact values whenever suitable.*
☒	☐	For Bayesian analysis, information on the choice of priors and Markov chain Monte Carlo settings
☒	☐	For hierarchical and complex designs, identification of the appropriate level for tests and full reporting of outcomes
☒	☐	Estimates of effect sizes (e.g. Cohen's *d*, Pearson's *r*), indicating how they were calculated

Our web collection on statistics for biologists contains articles on many of the points above.

Software and code

Policy information about availability of computer code

Data collection	Cryo-ET data were collected by Thermo Scientific Tomography 5 Software.
Data analysis	Cryo-ET data were processed by MotionCor2 version 1.5.0, CTFFIND4, IMOD version 4.11, eTomo, RELION4, emClarity, Chimera version 1.18, ChimeraX version 1.8, MemBrain, EMAN2, IsoNet_v0.2, GraphPad Prism 10, Image J.

For manuscripts utilizing custom algorithms or software that are central to the research but not yet described in published literature, software must be made available to editors and reviewers. We strongly encourage code deposition in a community repository (e.g. GitHub). See the Nature Portfolio guidelines for submitting code & software for further information.

Data

Policy information about availability of data

All manuscripts must include a data availability statement. This statement should provide the following information, where applicable:

- Accession codes, unique identifiers, or web links for publicly available datasets
- A description of any restrictions on data availability
- For clinical datasets or third party data, please ensure that the statement adheres to our policy

All data needed to evaluate the conclusions in the paper are present in the paper and/or the Supplementary information and source data are provided with this paper, EM maps are available in the public database EMDB with accession number: EMD-51333 and EMD-51334.

Research involving human participants, their data, or biological material

Policy information about studies with human participants or human data. See also policy information about sex, gender (identity/presentation), and sexual orientation and race, ethnicity and racism.

Reporting on sex and gender	Not applicable
Reporting on race, ethnicity, or other socially relevant groupings	Not applicable
Population characteristics	Not applicable
Recruitment	Not applicable
Ethics oversight	Not applicable

Note that full information on the approval of the study protocol must also be provided in the manuscript.

Field-specific reporting

Please select the one below that is the best fit for your research. If you are not sure, read the appropriate sections before making your selection.

☒ Life sciences ☐ Behavioural & social sciences ☐ Ecological, evolutionary & environmental sciences

For a reference copy of the document with all sections, see nature.com/documents/nr-reporting-summary-flat.pdf

Life sciences study design

All studies must disclose on these points even when the disclosure is negative.

Sample size	Sample sizes of tomograms are listed in Supplemental Table 1 and 2, and the sample sizes for subtomogram averaging are listed in Supplemental Table 2.
Data exclusions	For Subtomogram averaging, particles were picked by emClarity, junk particles were excluded after 3D classification.
Replication	For subtomogram averaging analysis, two randomly divided half datasets were processed independently and combined to give rise to the final structures. The resolution of the structure is assessed by comparing the two independent maps
Randomization	Subtomogram average of SARS-CoV-2 Victoria strain spike with or without WS6 bound, the dataset were randomly divided into two half sets, as a standard approach implemented in Relion
Blinding	The investigators were not blinded during the experiments or outcome assessment.

Reporting for specific materials, systems and methods

We require information from authors about some types of materials, experimental systems and methods used in many studies. Here, indicate whether each material, system or method listed is relevant to your study. If you are not sure if a list item applies to your research, read the appropriate section before selecting a response.

Materials & experimental systems

n/a	Involved in the study	
☐	☒	Antibodies
☐	☒	Eukaryotic cell lines
☒	☐	Palaeontology and archaeology
☒	☐	Animals and other organisms
☒	☐	Clinical data
☒	☐	Dual use research of concern
☒	☐	Plants

Methods

n/a	Involved in the study	
☒	☐	ChIP-seq
☒	☐	Flow cytometry
☒	☐	MRI-based neuroimaging

Antibodies

Antibodies used	anti-ACE2 antibody (66699-1-Ig, Proteintech; 1:5000 dilution) , an anti-mouse HRP-conjugated secondary antibody (A0168, Sigma-

Antibodies used	Aldrich; 1:5000 dilution), a monoclonal anti-S2 antibody (Thermo Fisher Scientific MA-5-35946) at a 1:4,000 dilution, a monoclonal anti-S2 antibody (Thermo Fisher Scientific MA5-42384; 1:5,000 dilution.
Validation	Each of these antibodies was previously validated and has been used in multiple published studies.

Eukaryotic cell lines

Policy information about cell lines and Sex and Gender in Research

Cell line source(s)	VERO C1008 [Vero 76, clone E6, Vero E6] cells were originally obtained from ATCC (USA), and the enti-X™ 293T Cell Line was purchased from Takara Bio (Japan)
Authentication	Cell lines were obtained from reputable commercial sources (ATCC and Takara Bio) and were not further authenticated. No cell lines used in this study were found in the ICLAC database of misidentified cell lines.
Mycoplasma contamination	N/A
Commonly misidentified lines (See ICLAC register)	N/A

Plants

Seed stocks	No plant used in this study.
Novel plant genotypes	No plant used in this study.
Authentication	No plant used in this study.
